# KDM4 inhibition alters H3K9me3 distribution and developmental responses in SETDB1-deficient embryonic stem cells

**DOI:** 10.64898/2026.10.14.758827

**Published:** 2026-10-16

**Authors:** Rui Geng, Benjamin L. Kidder

## Abstract

SETDB1 contributes to histone H3 lysine 9 methylation, whereas KDM4 demethylases act on H3K9 and, for KDM4A-C, H3K36. We examined whether Setdb1 status modifies responses to the KDM4-family inhibitor QC6352 in embryonic stem cells (ESCs) and embryoid bodies (EBs). QC6352 shifted Setdb1-depleted ESC colonies toward more compact morphology and increased alkaline-phosphatase (AP) staining, but also affected Setdb1-intact cells. Separate Kdm4a, Kdm4b, and Kdm4c knockdowns identified overlapping and member-specific transcriptional responses. Factorial RNA-seq revealed broadly concordant transcriptional responses to KDM4 inhibition in Setdb1-intact and depleted ESCs, alongside a smaller subset of genes with genotype-dependent treatment effects. Within this subset, two gene modules showed attenuation or reversal of Setdb1-associated expression changes following KDM4 inhibition. ChIP-seq revealed changes in the relative distribution of H3K9me3, H3K36me3, and H3K4me3 between promoters and gene bodies following KDM4 inhibition. KDM4 inhibition altered H3K9me3 distributions across repeat families in both Setdb1-intact and depleted ESCs. QC6352 also altered EB morphology and produced transcriptional responses that differed between ESCs and EBs. These findings show that KDM4 inhibition remodels chromatin and transcription in both Setdb1-intact and depleted ESCs, with Setdb1 status influencing a subset of transcriptional responses.

